# The influence of breast cancer subtype on survival after palliative radiation for osseous metastases

**DOI:** 10.1002/cam4.3597

**Published:** 2020-11-04

**Authors:** Mohamed K. Abdelhakiem, Candice Johnstone, Carmen Bergom, Adam Currey, Jared R. Robbins

**Affiliations:** ^1^ Department of Radiation Oncology University of Pittsburgh UPMC Hillman Cancer Center Pittsburgh PA USA; ^2^ Department of Radiation Oncology Medical College of Wisconsin Milwaukee WI USA; ^3^ Department of Radiation Oncology University of Arizona Tucson AZ USA

**Keywords:** breast cancer, osseous metastases, outcomes, palliative, radiation, subtype

## Abstract

**Background:**

Among patients with osseous metastases, breast cancer (BC) patients typically have the best prognosis. In the palliative setting, BC is often considered a single disease, but based on receptor status there are four distinct subtypes: luminal A (LA), luminal B (LB), triple negative (TN), and HER2‐enriched (HER2). We hypothesize that survival and palliative outcomes following palliative RT for osseous metastases correlate with breast cancer subtype (BCS).

**Methods:**

We identified 3,895 BC patients with known receptor status who received palliative RT for osseous metastases from 2004–2013 in the National Cancer Database. Kaplan–Meier method with log‐rank testing and univariate/multivariate Cox‐regression was used to identify survival factors. Incomplete radiation courses, 30‐day mortality rate, and percentage remaining life spent receiving RT (PRLSRT) were calculated.

**Results:**

Subtypes were 54% LA, 33% LB, 8% TN, and 5% HER2 with median survival of 34.1, 28.2, 5.3, and 15.7 months, respectively (*p* < 0.001). Overall 82% of patients received ≥10 fractions. Although BCS had limited effect on radiation regimens, TN received nearly twice as many single or hypofractionated (≤5 fractions) treatments, but the overall rate of these fraction schemes was low at 3.7 and 13.7%, respectively. Compared to LA and LB, TN and HER2 patients had worse palliative outcomes; higher rates of incomplete courses at 18.8% and 18.3% versus 12.7%–14.4%; higher 30‐day mortality post‐radiotherapy at 21.5% and 16.0% versus 6.3%–7.9%, and higher median PRLSRT of 7.7% and 3.7% versus 2.2%–2.4% for LA and LB. On multivariate analysis, BCS was associated with overall survival with TN (HR 3.7), HER2 (HR 1.75), and LB (HR 1.28) fairing worse than LA (*p* < 0.001).

**Conclusions:**

BCS correlated with survival and palliative outcome following radiation to osseous metastases. BCS should be considered by physicians when planning palliative RT to maximize quality‐of‐life, avoid unnecessary treatment, and ensure palliative benefits.

## Graphical abstract

This study demonstrates the impact of breast cancer subtype on survival and palliative outcomes after palliative radiation for osseous metastases. Patients with triple negative and HER2‐enriched subtypes had shorter survival, higher 30 and 90‐day mortality, had more incomplete treatments, spent more of their remaining lives receiving radiation treatments, but received similar radiation regimens compared to Luminal A and Luminal B patients. This correlation should be considered when selecting palliative radiotherapy regimens to avoid unnecessary treatment and improve palliative outcomes for poor‐prognosis patients.

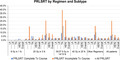

## 1 INTRODUCTION

Patients with bone metastases from breast cancers typically have a better prognosis than those with bone metastasis originating from other primary sites,^1^, ^2^ and therefore are often treated with longer, more intense radiation courses.^3^ However, breast cancer encompasses a spectrum of distinct subtypes with varying prognosis.^4^ Traditionally, breast cancer has been classified into four distinct subtypes: luminal A (most common, best prognosis), luminal B, triple negative (worst prognosis), and Her2‐enriched.^5^, ^6^ Although breast cancer subtype (BCS) strongly determines primary treatment, there is little published on how BCS affects palliative radiotherapy regimens. Understanding these relationships is critical for determining the most appropriate dose fractionation scheme for each patient. We hypothesize that BCS predicts overall survival following palliative radiotherapy for osseous metastases and that the subtypes with poorer prognosis will also have inferior palliative outcomes as measured by 30‐day post‐RT mortality, number of incomplete radiation courses, and the percentage of remaining life spent receiving radiation therapy. To examine this hypothesis, we analyzed breast cancer patients receiving palliative radiation to osseous metastases from the National Cancer Database.

## 2 METHODS AND MATERIALS

This Institutional Review Board approved‐study surveyed the National Cancer Database (NCDB) from years 2004 to 2013 to identify patients with osseous metastases from breast cancer through the utilization of the International Classification of Diseases‐Third revision (ICD‐3) site‐specific code, C500‐509 (breast), and the radiation treatment volume for bone (24–28, 37, 38). Radiation dose and fractionation were evaluated, and doses not consistent with palliative bone radiation were excluded. Breast cancer subtype was determined using receptor status and tumor grade from the database to categorize patients as defined^7^, ^8^, ^9^, ^10^:

1. Luminal A (LA): ER+and/or PR+, HER2‐
2. Luminal B (LB): ER+and/or PR+, HER2+; or ER+and/or PR+, HER2‐ with tumor grade 3
3. Triple negative (TN): ER‐, PR‐, HER2‐
4. HER2‐enriched (HER2): ER‐, PR‐, HER2+

The Kaplan–Meier method with log‐rank testing was used to evaluate survival outcomes. Univariate analysis was performed to identify variables associated with survival, with significant variables (*p* < 0.05) being included in a stepwise Cox‐regression model. Survival was calculated from the start of radiation therapy to the time of death or last follow‐up. Three metrics of palliative radiation quality were evaluated: the number of incomplete treatments, the 30‐day post RT mortality, and the percentage of remaining life patients spent receiving radiation therapy (PRLSRT). PRLSRT was calculated using the elapsed days of radiation (calendar days from start to completion) divided by the time from the start of radiation to death or last follow‐up. Treatment courses were considered to be incomplete if a standard palliative dose per fraction (8 Gy, 5 Gy, 4 Gy, 3 Gy, 2.5 Gy, and 2 Gy) were used, but the total dose was not equal to a common palliative regimen total for 40 Gy, 37.5 Gy, 35 Gy, 30 Gy, 25 Gy, 20 Gy, or 8 Gy. This method for determinizing incomplete treatment has been reported by others.^11^

## 3 RESULTS

**FIGURE 1:**
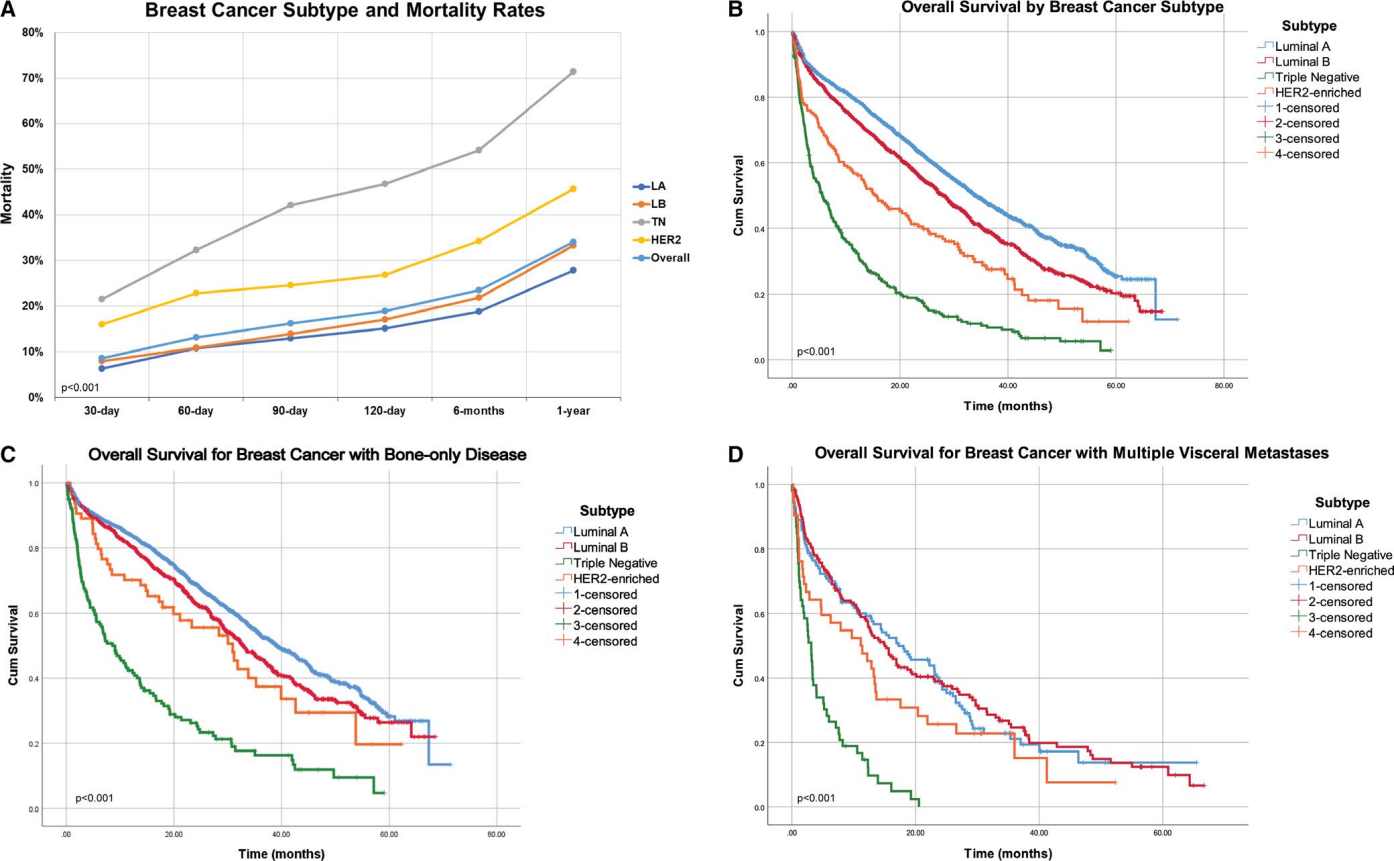
Mortality and survival. A, Mortality rate by breast cancer subtype. B–D, Overall survival by breast cancer subtype. B, Entire population. C, Patients with bone‐only disease. D, Patients with multiple visceral metastases. LA indicates luminal A; LB, luminal B; TN, triple negative

A total of 3895 breast cancer patients, who received palliative radiotherapy for osseous metastases from 2004–2013 were identified in the NCDB. Patient characteristics are described in Table 1. Mean patient age was 61 years with a range of 21–90 years. Median follow‐up was 20.4 months. LA was the most common subtype at 54.6% followed by LB at 33%, TN at 7.9%, and HER2 at 4.3%. Both HER2 and TN patients were more likely than either LA or LB patients to receive cytotoxic chemotherapy and to present with liver metastases or multisite visceral metastases. HER2 and TN patients had worse survival; the median overall survival from the start of radiotherapy was 5.3 months for TN and 15.7 months for HER2 compared to 34.1 and 28.2 months for LA and LB, respectively (*p* < 0.001) (Figure 1). Over half of all TN patients and 34% of HER2 patients were deceased by 6 months. (Figure 1). For patients with bone‐only metastases, breast cancer subtype remained predictive of survival with median overall survival for LA of 39.1 months, LB of 32.9 months, TN of 8.6 months, and HER2 of 30.9 months (overall *p* < 0.001, by pairwise comparison all relationships remained significant except LB and HER2 (*p* = 0.181) (Figure 1.).

**TABLE 1:** Characteristics of breast cancer patients receiving palliative RT

Variables (n)	Total cohort (n = 3922)	Subtype				
		Luminal A (n = 2126, 54.2%)	Luminal B (n = 1296, 33%)	Triple Negative (n = 315, 8.3%)	HER2 (n = 168, 4.5%)	*p*‐value
Age (y), median (range)	61 (21–90)	63 (22–90)	59 (21–90)	61 (25–90)	58 (27–90)	*p* < 0.001
Race						
Caucasian	3192 (81.4%)	1798 (84.6%)	1007 (77.7%)	246 (75.7%)	141 (80.5%)	*p* < 0.001
African American	583 (14.9%)	256 (12%)	233 (18%)	70 (21.6%)	24 (13.7%)	
Others	95(2.4%)	48 (2.3%)	38 (2.9%)	4 (1.2%)	5 (2.9%)	
Unknown	52 (1.3%)	24 (1.1%)	18 (1.4%)	5 (1.5%)	5 (2.9%)	
Insurance						
Not insured	251 (6.4%)	131 (6.2%)	87 (6.7%)	21 (6.7%)	12 (7.1%)	*p* = 0.038
Private	1560 (40%)	799 (37.6%)	558 (43.1%)	128 (40.6%)	75 (45.2%)	
Government	2034 (52.1%)	1162 (54.6%)	634 (48.9%)	162 (51.4%)	76 (44.6%)	
Unknown	60 (1.6%)	34 (1.6%)	17 (1.3%)	4 (1.3%)	5 (3.0%)	
Median Income Quartile						
1st	709 (18.2%)	346 (16.3%)	262 (20.2%)	72 (23.2%)	29 (17.4%)	*p* = 0.025
2nd	851 (21.8%)	466 (21.9%)	277 (21.4%)	75 (24.1%)	33 (19.8%)	
3rd	1131 (28.9%)	623 (29.3%)	367 (28.3%)	87 (28.0%)	54 (32.3%)	
4th	1189 (30.4%)	680 (32%)	381 (29.4%)	77 (24.8%)	51 (30.5%)	
Charlson‐Deyo Score						
0	3159 (80.9%)	1722 (81%)	1048 (80.9%)	250 (79.4%)	139 (82.9%)	*p* = 0.536
1	553 (14.2%)	309 (14.5%)	181 (14%)	45 (14.3%)	18 (10.7%)	
≥2	193 (4.9%)	95 (4.5%)	67 (5.1%)	20 (6.3%)	11 (8.3%)	
Metastases at diagnosis						
None	155 (4%)	75 (3.5%)	48 (3.7%)	26 (8.3%)	6 (3.6%)	*p* < 0.001
Osseous only	2375 (60.8%)	1434 (67.5%)	743 (57.3%)	135 (42.9%)	63 (37.5%)	
Brain any	103 (2.6%)	56 (2.6%)	31 (2.4%)	8 (2.5%)	8 (4.4%)	
Liver any	392 (10.0%)	160 (7.5%)	147 (11.4%)	47 (14.9%)	38 (16.9%)	
Lung any	490 (12.5%)	273 (12.9%)	157 (12.1%)	48 (15.2%)	11 (6.5%)	
Multiple Visceral organs	391 (10%)	128 (6%)	170 (13.1%)	51 (16.2%)	42 (25%)	
Osseous treatment sites						
Axial	2748 (70.4%)	1480 (69.6%)	918 (70.8%)	222 (69.8%)	128 (76.2%)	*p* = 0.328 *p* < 0.001
Appendicular	1157 (29.6%)	646 (30.4%)	378 (29.2%)	93 (30.2%)	40 (23.8%)	
Spine	2638 (67.2%)	142 (66.8%)	880 (67.9%)	209 (64.3%)	129 (73.7%)	
Skull	55 (1.4%)	26 (1.2%)	20 (1.6%)	7 (2.2%)	2 (1.2%)	
Ribs	65 (1.7%)	34 (1.6%)	18 (1.4%)	11 (3.4%)	2 (1.2%)	
Hip	505 (12.9%)	268 (12.6%)	165 (12.7%)	47 (14.4%)	25 (14.2%)	
Pelvic	251 (6.4%)	143 (6.7%)	83 (6.4%)	18 (5.5%)	7 (4%)	
Shoulder	93 (2.4%)	53 (2.5%)	31 (2.4%)	7 (2.2%)	2 (1.2%)	
Extremities	315 (8%)	182 (8.6%)	99 (7.6%)	26 (8%)	8 (4.5%)	
Chemotherapy						
No	2211 (56.6%)	1534 (72.1%)	560 (43.2%)	90 (28.6%)	27 (16.1%)	*p* < 0.001
Yes	1619 (41.5%)	544 (25.6%)	718 (55.4%)	219 (69.5%)	138 (82.1%)	
Unknown	75 (1.9%)	48 (2.3%)	18 (1.4%)	6 (1.9%)	3 (1.8%)	
Hormone therapy						
No	1080 (27.7%)	285 (13.4%)	328 (25.3%)	304 (99.0%)	163 (98.2%)	*p* < 0.001
Yes	2758 (70.6%)	1808 (85%)	940 (72.5%)	0 (0%)	0 (0%)	
Unknown	67 (1.7%)	33 (1.6%)	28 (2.2%)	3 (1.0%)	3 (1.8%)	
Median PRLSRT	2.5%	2.1%	2.4%	8.3%	3.8%	

The most common radiotherapy regimen was 30 Gy in 10 fractions regardless of subtype. Advanced radiation techniques were used infrequently (3% Intensity Modulated Radiation Therapy, 1% receiving stereotactic body radiation therapy). The median duration of radiotherapy was 15 days. Insurance status correlates with receiving extended fractions (≥10): private 84.0%, government 81.2%, none 79.3%, and unknown 75.4% (*p* = 0.015). Table 2 shows the distributions of treatment regimens by breast cancer subtype. Overall, there was a preference for shorter courses for TN and HER2 subgroups with TN patients receiving nearly double the number of single fraction or hypofractionated (≤5 fractions) regimens. Despite the increased proportional use of fewer fractions for the TN and HER subtypes, overall single fraction treatments were used infrequently (3.7% of total). Overall 82% of all patients received ≥10 fractions (83.1% for LA, 81.9% for LB, 68.6% for TN, and 78.3% for HER2). About 14.0% of all patients stopped radiation early; the rate of incomplete treatments was significantly higher for TN (18.8%) and HER2 (18.3%) versus LA (12.7%) and LB (13.4%) (*p* < 0.001) (Table 3). Factors affecting the rate of incomplete radiation treatment include Charlson‐Deyo comorbidity index (13.6% for score 0, 12.6% for score 1, 23.8% for score 2, *p* < 0.001), osseous location (8.9% for appendicular vs. 16.1% for axial, *p* < 0.001), dose per fraction (0% for 8 Gy, 3.8% for 4 Gy, 24.6% for 3 Gy, 38.0% for 2.5 Gy and 12.5% for 2 Gy, *p* < 0.001), and breast cancer subtype (12.7% for LA, 14.4% for LB, 18.8% for TN, and 18.3% for HER2, *p* = 0.007). Overall the 30‐day and 90‐day post‐RT mortality was 8.5% and 16.2%, respectively. During these periods, the mortality rate for HER2 (16.0%; 24.6%) and TN (21.2%; 42.2%) was 2–3 times higher than LA and LB patients (6.3%–7.9%; 12.9%–14.0%) (*p* < 0.001).

**TABLE 2:** Radiation treatment regimen distribution by subtype and fractionation median PRLSRT

Radiation Regimen	Overall (% Patients)	Luminal A (% Patients)	Luminal B (% Patients)	Triple Negative (% Patients)	HER2‐enriched (% Patients)	Median PRLSRT
40 × 20	1.6%	1.8%	1.2%	1.8%	1.7%	3.8%
37.5 × 15	9%	9.1%	10%	6.2%	5.1%	2.9%
35 × 14	11.3%	12.4%	10.3%	7.7%	12%	3%
30 × 10	49.3%	49.6%	50.2%	43.1%	50.9%	2.4%
25 × 5	0.2%	0.2%	0.2%	0.3%	0%	1.6%
20 × 5	5.1%	4.7%	4.9%	9.2%	4%	1.4%
8 × 1	2.4%	2.4%	1.8%	4.6%	2.9%	0.2%
Other	7.1%	7.1%	7%	8.3%	5.1%	2.5%
Incomplete	14%	12.7%	14.4%	18.8%	18.3%	3.9%
Single fraction	3.7%	3.6%	3%	6.5%	4.6%	0.2%
2–5 fractions	10.0%	9.4%	9.2%	17.8%	9.1%	1.5%
6–10 fractions	54.3%	53.6%	56.1%	50.2%	58.9%	2.4%
>10 fractions	32%	33.5%	31.7%	25.5%	27.4%	3.1%
Hypofractionated						
(1–5 fx)	13.7%	13.0%	12.2%	24.3%	13.7%	1.2%

**TABLE 3:** Number of complete versus incomplete palliative radiation therapy courses by subtype

Subtype	Completed RT Course	Incomplete RT Course	% Incomplete RT Course	Median Overall Survival (months)	Complete Median Survival (months)	Incomplete Median Survival (months)	Complete Median PRLSRT	Incomplete Median PRLSRT	Overall Median PRLSRT
Luminal A	1857	269	12.7%	34.1	36	18.9	2.2%	3%	2.2%
Luminal B	1109	187	14.4%	28.2	29	15.7	2.4%	3.9%	2.4%
Triple negative	255	60	19.0%	5.3	7.1	1.2	6.4%	25.8%	8.6%
HER2‐enriched	136	32	19.0%	15.7	19.9	8.1	3.4%	7.0%	3.7%
Overall	3357	548	14%	28.5	30.3	13.9	2.4%	3.9%	2.5%

**FIGURE 2:**
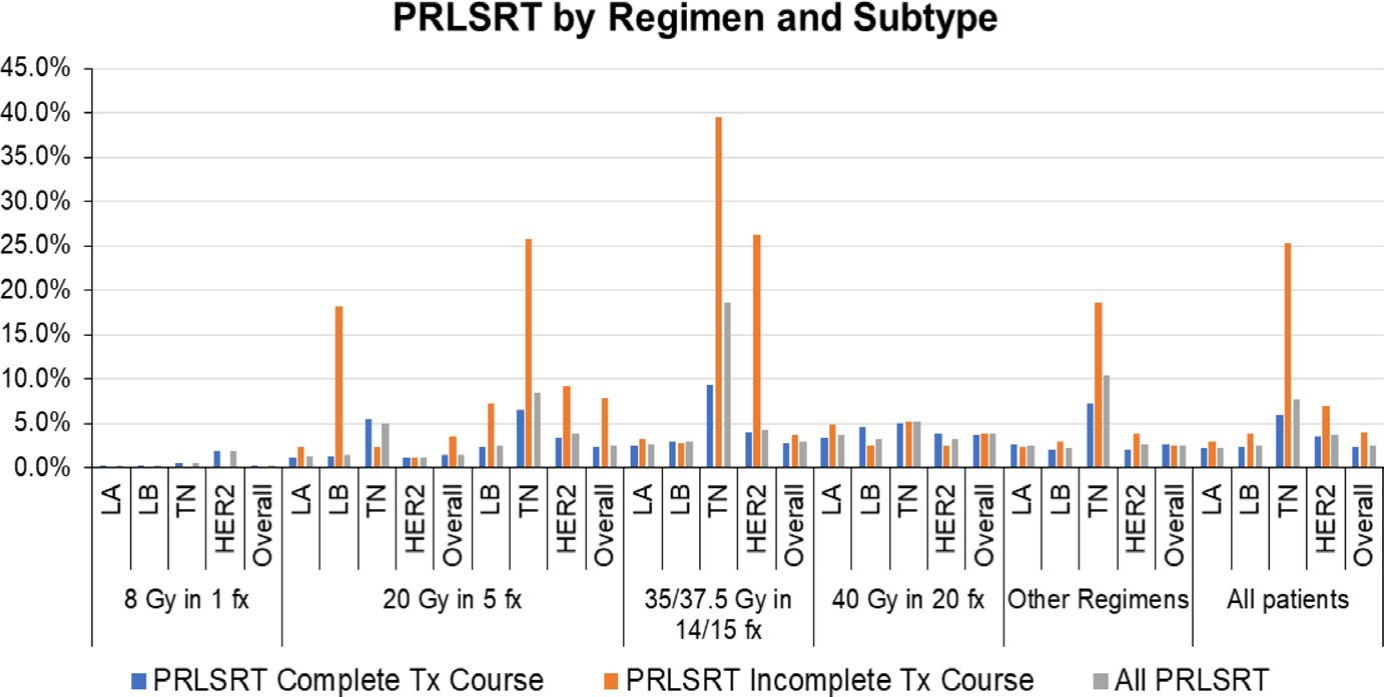
Percentage remaining life spent receiving treatment (PRLSRT) of alive and deceased patients by subtype. LA indicates luminal A subtype; LB, luminal B; TN, triple negative

Overall 4.4% of patients had a PRLSRT of >50%, 10% had a PRSLRT of >25% and 19.4% had a PRLSRT of >10%. PRLSRT was impacted by multiple variables most notably the length of treatment, the completion of treatment, and the breast cancer subtype (Figure 2). Analysis of variables associated with 10%, 25%, and 50% PRLSRT identified multiple significant variables (Table 4). Of note, PRLSRT of ≥50% was observed in 13.2% of TN patients, 9.7% of HER2 patients, 14.6% of patients with incomplete RT courses, and 11.9% of those with Charlson‐Deyo score of 2.

**TABLE 4:** Relationships of patient characteristics and PRLSRT (10%, 25%, and 50%)

Category	10% PRLSRT	25% PRLSRT	50% PRLSRT	*p*‐value
Overall (all patients)	19.4%	10.0%	4.4%	—
Age				*p* < 0.001
≤65	15.4%	7.8%	3.5%	
>65	26.5%	13.8%	6.1%	
Charlson‐Deyo score				*p* < 0.001
0	18.2%	9.5%	3.9%	
1	22.5%	10.6%	5.0%	
2	31.1%	17.6%	11.9%	
Incomplete RT				*p* < 0.001
No	16.8%	7.5%	2.8%	
Yes	35.7%	25.7%	14.6%	
Metastases at diagnosis				*p* < 0.001
Osseous only	14.2%	6.7%	2.8%	
Lung	24.4%	15.3%	6.3%	
Brain	27.9%	11.5%	5.8%	
Liver	26.0%	14.6%	7.3%	
None	23.7%	12.2%	5.1%	
Multiple visceral sites	34.1%	17.6%	8.7%	
Number of fractions				*p* < 0.001
1 fraction	6.3%	1.4%	0.7%	
2–5 fractions	24.7%	14.8%	8.2%	
6–10 fractions	19.8%	9.8%	4.4%	
>10 fractions	18.6%	9.9%	3.7%	
Osseous site of metastasis				*p* < 0.001
Appendicular	14.4%	6.6%	0.7%	
Axial	21.5%	11.5%	3.7%	
BC subtype				*p* < 0.001
LA	15.8%	7.8%	2.8%	
LB	17.5%	8.8%	4.2%	
TN	45.8%	25.5%	13.2%	
HER2	29.1%	17.7%	9.7%	

Multivariate Cox‐regression analysis identified breast cancer subtype, age, private insurance status, metastatic site at diagnosis, treatment of axial skeletal metastases, Charlson‐Deyo comorbidity score, and the number of radiation treatments was significant predictors of overall survival (Table 5). As the aggressiveness of the breast cancer subtype increased so did the risk of death with all other subtypes being worse than LA, and TN patients having almost four times the risk of death compared to LA. Similar analysis limited to bone‐only disease confirmed the significance of BCS for survival (Hazard Ratio: LA (ref), LB 1.322 (1.165–1.504, *p* < 0.001), TN 4.092 (3.220–5.043, *p* < 0.001), HER2 1.589 (1.129–2.237, *p* = 0.008) (Figure 1).

**TABLE 5:** Cox‐regression analysis of overall survival

Category	Median Survival (months)	Univariate Analysis			Multivariate Analysis		
		Hazard Ratio	95% confidence interval	*p*‐value	Hazard Ratio	95% confidence interval	*p*‐value
Age, continuous variable	—	1.017	1.013–1.020	0.001	1.015	1.012–1.019	<0.001
Charlson‐Deyo score							
0	30.6	(ref)	(ref)	(ref)	(ref)	(ref)	(ref)
1	21.1	1.350	1.204–1.514	<0.001	1.230	1.095–1.382	0.001
2	11.6	1.902	1.596–2.267	<0.001	1.621	1.356–1.936	<0.001
Race							
White	28.9	(ref)	(ref)	(ref)	(ref)	(ref)	(ref)
African American	24.3	1.163	1.036–1.306	0.01	1.045	0.928–1.177	0.467
Other	38.1	0.766	0.568–1.033	0.081	0.654	0.483–0.886	0.006
Unknown	38.9	0.176	0.506–1.132	0.757	0.717	0.478–1.067	0.109
Insurance status							
Private	34.9	(ref)	(ref)	(ref)	(ref)	(ref)	(ref)
Government	23.3	1.473	1.348–1.611	<0.001	1.267	1.144–1.402	<0.001
Uninsured	24.5	1.366	1.142–1.636	<0.001	1.274	1.062–1.529	0.009
Unknown	17.7	1.554	1.118–2.161	<0.009	1.487	1.067–2.070	0.019
Metastases at diagnosis							
Osseous only	35.0	(ref)	(ref)	(ref)	(ref)	(ref)	(ref)
Lung	24.1	1.578	1.392–1.789	<0.001	1.468	1.294–1.666	<0.001
Brain	19.5	2.088	1.655–2.634	<0.001	1.989	1.5673–2.515	<0.001
Liver	18.3	1.807	1.581–2.064	<0.001	1.788	1.560–2.049	<0.001
None	24.1	1.324	1.324–1.069	0.010	1.185	0.952–1.476	0.128
Multiple visceral sites	12.4	2.445	2.163–2.786	<0.001	2.344	2.055–2.673	<0.001
Number of fractions							
1 fraction	24.4	1.435	1.149–1.792	0.001	1.349	1.075–1.693	0.010
2–5 fractions	17.7	1.766	1.529–2.040	<0.001	1.585	1.370–1.834	<0.001
6–10 fractions	27.7	1.178	1.072–1.294	0.001	1.134	1.031–1.247	0.010
>10 fractions	32.0	(ref)	(ref)	(ref)	(ref)	(ref)	(ref)
Osseous site of metastasis							
Appendicular	30.0	(ref)	(ref)	(ref)	(ref)	(ref)	(ref)
Axial	27.6	1.121	1.022–1.230	0.015	1.165	1.060–1.280	0.001
Breast cancer subtype							
Luminal A	34.1	(ref)	(ref)	(ref)	(ref)	(ref)	(ref)
Luminal B	28.2	1.267	1.154–1.391	<0.001	1.282	1.165–1.411	<0.001
Triple Negative	5.3	3.816	3.338–4.364	<0.001	3.892	3.226–4.479	<0.001
HER2‐enriched	15.7	1.931	1.603–2.326	<0.001	1.747	1.437–2.124	<0.001

## 4 DISCUSSION

Breast cancer patients who develop bone metastases are generally thought to have a favorable prognosis compared to those with bone metastases from other cancer types.^1^, ^2^, ^3^ Breast cancer is a favorable prognostic factor for survival after palliative radiotherapy in two popular prognostic models.^1^, ^12^ In these models, the hazard ratio for overall survival was roughly 30–90% lower for breast cancer compared to prostate cancer, the second most favorable cancer, and significantly lower for other cancers. A recent Danish study showed the 3‐year overall survival after diagnosis of bone metastases to be significantly higher for breast at 25% compared to 12% for prostate, 10% for renal, 7% for colon, 3% for rectum, and 2% for lung.^3^ While the current study reports a favorable survival outcome for the entire breast cancer cohort (3‐year overall survival of 41%), there are significant differences in survival outcomes based on receptor status (3‐year overall survival of 47% for LA, 39.2% for LB, 28.1% for HER2, and 8.3% for TN). A similar worse prognosis for TN was also observed in the cohort with bone‐only metastases. These disparate outcomes suggest that breast cancer subtypes should be considered separately when determining prognosis after palliative radiotherapy.

The differences in survival outcomes between breast cancer subtypes are likely multifactorial.^9^ Despite limited data evaluating the impact of BCS on survival after palliative radiotherapy for bone metastases, worse survival rates for TN and HER2 cases have been observed in the localized and metastatic setting with these having a two to fourfold increased risk of death and a one‐half to one‐third shorter median survivals compared to LA.^10^, ^13^ Additionally each subtype tends to have a preference for the metastatic site. LA and LB subtypes have a predilection for bone‐only metastases, while TN and HER2 have higher incidence of brain, liver, and lung metastases.^14^, ^15^ The higher propensity for visceral metastases in TN and HER2 subtypes likely contribute to their worse prognosis as visceral metastases are known risk factors for death.^16^ The effectiveness of systemic therapies for each subtype likewise contributes to the differences in outcome with ER/PR receptor positive subtypes being very responsive to antihormonal therapy and HER2 to targeted therapies.

The goals of palliative radiation to osseous metastases are symptom management, maintaining the quality of life, and preventing tumor progression, with pain control being the most common and often overarching goal. Roughly 25 randomized trials have shown that short courses of RT are equally effective at pain control compared to longer multifraction courses. The notion that breast cancer patients with bone metastases has better prognosis, often results in prolonged radiation treatments. This is borne out in the present study with more than 82% of patients prescribed ≥10 fractions. Although TN and HER2 patients received a slightly higher proportion of short treatment courses, the overall utilization of single and hypofractionated treatments was limited to 4% and 13%, respectively. Although the number of fractions for radiation treatments was correlated with survival on multivariate analysis in this study, this is likely a reflection of patient selection and patients with the worst prognosis not completing their treatments rather than an actual improved response with increasing dose, especially since the most common indication for radiation is pain control. For the most part, extended treatments are problematic for patients with poor prognosis, leading to a disproportionally larger portion of their remaining life spent receiving treatment and higher rates of incomplete courses. One metric to evaluate the appropriateness and length of the palliative radiation treatment is the post‐RT mortality at 30 and 90 days, which identifies those who die shortly after radiation delivery and subsequently may not have benefited from palliative RT, since the median time to pain response is 3 weeks.^17^ The 30 and 90‐day mortality rate for HER2 and TN was 2–3 times higher than LA and LB patients, suggesting that many HER2 and TN patients may not have lived long enough to derive benefit from radiation. (Figure 1).

Although the choice of fractionation scheme may seem arbitrary, the length of treatment can significantly affect the palliative outcome. The PRLSRT reflects the burden of treatment.^18^ Patients with a high PRLSRT had a high treatment burden, little time to derive the anticipated palliative benefits, and likely a net detriment to quality‐of‐life, well‐being, and resources. In this sense, PRLSRT forces physicians to consider how decisions regarding treatment length, the patient's estimated prognosis, and the time required to achieve the desired palliative goal impact how patients spend their remaining days—spending time with loved ones or commuting for treatment. In this study, higher PRSLRT was observed with longer RT courses, more aggressive breast cancer subtypes, visceral metastases, and incomplete treatment (Table 4). As a quality metric for palliative radiation, PRLSRT should be less than 10%;^19^ meaning the duration of RT is less than 10% of the time from the start of radiotherapy to death. In the present study, about 20% of patients had a PRLSRT of at least 10%, but the rate was much higher in the TN and HER2 cohort at about 45% and 30%, respectively (Table 4). These groups also had a relatively high rate of PRLSRT ≥50% (13% and 9%, respectively), which again highlights the potential benefit of a shorter course of treatment in this subgroup, particularly given the equipoise in pain response and less cost for short courses.^12^, ^20^

Failure to complete a prescribed course of palliative radiotherapy can signify that the course was too long, the pretreatment prognosis was overly optimistic, or the anticipated benefits were not worth the expense of time and resources.^21^, ^22^ There is little data regarding the frequency of incomplete courses of palliative radiotherapy aside from the end‐of‐life setting, which shows high rates of incomplete radiation (42%–72%) with most patients stopping treatment secondary to death, hospice enrollment, or patient preference.^18^, ^22^, ^23^ Limited data from retrospective and prospective studies report incompletion rates of 7%–10%.^21^, ^24^ In the current study, the rate of incomplete radiation was 14% with higher rates in patients with higher Charlson‐Deyo score, treatment of axial bone, longer treatment course, and TN and HER2 breast cancer subtype. The present study also confirms the findings of others that incomplete treatment was associated with poor survival outcomes and higher PRLSRT.^18^, ^21^, ^22^, ^25^ For these reasons, incomplete treatment is considered a quality metric for palliative radiotherapy.^19^ Based on the available data consideration of patient morbidity, performance status, treatment site, and BCS can avoid incomplete treatment courses.

Quality‐of‐life and reduced suffering are two defining tenets of palliative radiotherapy. It is therefore essential to consider prognostic factors in order to personalize treatment courses and provide therapy that matches patient's goals and prognosis. Due to the significant impact breast cancer subtype has on survival and palliative outcomes, including it in prognostic calculations will likely improve treatment selection for all patients. As such poor‐prognosis patients could preferably benefit from short courses, which provide equal pain response as longer courses, but allow patients more time to spend as they wish.^12^, ^20^ Likewise, good prognosis patients may benefit from intense courses given some emerging benefits of more aggressive radiotherapy in good prognosis patients and are the main question in the ongoing NRG BR002 study.^17^, ^26^ Currently there is limited data of differing responsiveness of the various breast cancer subtypes to palliative radiation, but several studies have confirm the improved survival for luminal types after palliative RT.^27^, ^28^ Likewise, this study does identify additional factors that can impact survival after palliative RT and should also be considered when determining the duration and intensity of RT. These include advanced age, multi‐organ metastatic disease, Charlson‐Deyo comorbidity score, and the axial bone metastases.

Limitations of this study include its retrospective design and reliance on the NCDB. The database does not include pathological fracture, soft tissue component or pain level, which could influence dose selection. It also lacks the level of ER/PR expression or detailed genotyping which precludes a more accurately define subtype, but the surrogates used in this study are commonly used in the literature.^29^ Careful efforts were taken to mitigate these limitations.

Each clinical subtype of breast cancer displays a unique behavior, metastatic tendency, and specific prognostic outcome. As such, receptor status is routinely used to tailor systemic therapy, but is infrequently applied to determining a palliative radiation prescription or prognosis after it. This reality is confirmed by the present study as the use of hormonal therapy and chemotherapies was strongly influenced by subtype, but there was a little difference in the radiation schemes, which led to worse palliative outcomes for TN and HER2 patients. It is unclear whether this disparity is related to the popularly applied prognostic models lumping all breast cancer patients together, the dearth of data relating survival after palliative radiotherapy based on receptor status, radiation oncologists being reluctant to use shorter course of treatment, or something else entirely. The present study may help rectify this situation by providing evidence for the inferior survival and palliative outcomes for TN and HER2 patients and helping physicians to better adapt radiation plans to the patient's actual prognosis, thereby providing better palliative outcomes and patients more time to spend as they wish. This study does not suggest that patients with TN or HER2 will not benefit from RT, but rather suggests we should be mindful when determining the duration and number of radiation treatments for these patients as to not erode the palliative benefit they will likely derive from them.

## 5 CONCLUSIONS

BCS is correlated with survival following palliative radiotherapy to osseous breast cancer metastases with strong correlation with three key metrics for evaluating palliative radiotherapy. Given the disparity in outcomes between subtypes, physicians should consider receptor status when choosing a palliative radiotherapy regimen to avoid excessive treatment and inconvenience, especially for poor‐prognosis patients. Weighing the benefits and risks of palliative radiotherapy based on receptor status is an important consideration for discussing treatment expectations, goals of care, and quality‐of‐life with these patients. Additional study is warranted to confirm and validate these findings.

## CONFLICT OF INTEREST

The authors declare that there is no conflict of interest.

## AUTHOR CONTRIBUTIONS

Contributor Roles/Conceptualization (JRR, MKA, CJ, AC). Contributor Roles/Data curation (JRR, MKA). Contributor Roles/Formal analysis (JRR, MKA). Contributor Roles/Investigation (JRR, MKA). Contributor Roles/Methodology (All). Contributor Roles/Resources (All). Contributor Roles/Software (JRR, MKA). Contributor Roles/Supervision (JRR, CJ, CB, AC). Contributor Roles/Validation (All). Contributor Roles/Visualization (All). Contributor Roles/Writing – original draft (JRR, MKA). Contributor Roles/Writing – review & editing (All).
